# Braithwaite’s Retrospect and Half-Yearly Abstract

**Published:** 1873-03

**Authors:** 


*Braithwaite's Retrospect and Half-Yearly Abstract*

Have been received. These publications should be in the hands of every doctor. The first is published by W. A. Townsend, 177 Broadway, New York; the latter by H. C. Lea, Nos. 106 and 108 Sansom street, Philadelphia.

